# Laundry and Sanitary Exhibition at the Agricultural Hall

**Published:** 1895-11-23

**Authors:** 


### LAUNDRY AND SANITARY EXHIBITION AT THE AGRICULTURAL HALL.

Messrs. Cordingley and Co., proprietors of *Laundry News*, are to be congratulated upon their successful organisation of the Laundry Exhibition opened at the Agricultural Hall, Islington, last week. Such an exhibition is of no small importance, collecting, as it does, under one roof all that is newest and best in the market in its special line, and enabling those interested in questions of public sanitation to see and judge for themselves as to the comparative merits of the most recent inventions.

There were many well-known names among those of the exhibitors, and many improvements might be noted in the various machinery since last year, of which space will allow but a passing notice. A new "Patent Combined Washer, Hydro-Extractor, Disinfector, and Tumbling Machine" (Mr. E. D. Reeve), a complete laundry in itself, in fact, has met with a good deal of approval, and attracted much attention. Messrs. Summerscales had an extensive show of their well-known specialities, the latest addition being the "Practical" washing, boiling, and disinfecting machine, made entirely of metal. The Blackman Ventilating Company, in addition to their fans and drying chambers, had on view a new "Special Air Warmer," with or without fan, for fixing over existing ironing stoves and utilising otherwise wasted steam for airing or drying rooms. The Liddell Engineering Company, Messrs. Potts, Cassell, and Williamson, Messrs. D. and J. Tullis, and Messrs. Watson, Laidlaw, and Co. exhibited hydro-extractors, washers, and ironing machine, while the Keighley Engineering Company showed their ventilated patent ironing machine, the perforations in the drum having a most preservative effect upon the covering felt. This firm had also on view three "New Patent Sunflower" washing and starching machines. It is claimed for the washer that its shape and construction conduce to an action more nearly resembling hand-washing than has been yet achieved. Messrs. Broadbent's well-known hydro-extractor might have been seen at work, and Messrs. James Armstrong seem to have transported the contents of several steam laundries into the large space reserved for their exhibits. The brass washer, in two compartments, shown by this firm, is a splendidly-made machine. Of water-softening apparatus there were several specimens, notably those of Messrs. Doulton and Co. and the Water Softening and Purifying Patent Company, showing the Tyacke and Slack and Brownlow's softeners. Soaps, starches, blues, and many other manufactures connected with laundry work were well represented. Many visitors have found their way to Islington during the past week. The development in laundry equipment of late years has been great, and in the case of hospitals and institutions where a large steam laundry forms an important part of the domestic economy, a keen desire that the methods there employed shall be of the best is beginning to animate those responsible for their management. To these the recent exhibition will have been of much interest.

